# Supplementary material for: The effects of proteasomal inhibition on synaptic proteostasis
Source: EMBO J. 2016 Sep 9;35(20):2238–62. doi: 10.15252/embj.201593594 (PMC5069550; doi:10.15252/embj.201593594)
Supplement: Supplementary file 1 — Appendix [file EMBJ-35-2238-s001.pdf]

Appendix to:

The Effects of Proteasomal Inhibition on Synaptic Proteostasis

Vicky Hakim, Laurie D. Cohen, Rina Zuchman, Tamar Ziv, Noam E. Ziv

Table of Contents:

Table of Contents	page 1
Supplementary Materials and Methods	page 2
Legends to Appendix Supplementary Figures S1 to S5	pages 3-4
Appendix Figures S1 to S5	pages 5-9

Supplementary Materials and Methods

Derivation of expected $log_2(H/M)$ values from known half-lives (equation 1 in main text)

Let us assume that at time *t=0*, a cell contains some initial amount of some protein and that the degradation of this protein occurs as a first order reaction with some fixed rate *r*. As a result, the fraction *f* of this initial amount remaining after some time *t* (as illustrated in Fig. 8A) is given by the expression

(1) $$f = e^{-t/\tau}$$

Where τ is the time constant of the degradation process. $\tau = 1/r$ and is proportional to the half-life ($t_{½}$) of the protein such that $\tau = log_2 e \cdot t_{½} \approx 1.443 \cdot t_{½}$.

Now let us assume that a pharmacological inhibitor reduces degradation rates by some factor α, resulting in an effective time constant of $\alpha\tau$ (Fig. 8A). In this case, the fraction *f* of some initial amount remaining after some time *t* is given by the expression:

(2) $$f = e^{-t/\alpha\tau}$$

In the experiments described in the main text, the major measurement was the H/M ratio, which reflects the residual amount of a particular protein pre-labeled with heavy AAs (H) after some time *t* in the presence of an inhibitor, divided by the residual amount of the same protein pre-labeled with medium AAs (M) in untreated neurons and the same *t*. At time 0, and after normalization, H ≈ M or in other words, the initial amounts of labeled variants of protein *k* are identical for control and inhibited conditions (Fig. 5B). Therefore, for a particular protein, we can refer to *f* in the presence of an inhibitor as f_H, and to *f* under control conditions as f_M and write

(3) $$\frac{H}{M} = \frac{f_H}{f_M} = \frac{e^{-t/\alpha\tau}}{e^{-t/\tau}} = e^{\frac{t(\alpha-1)}{\alpha\tau}}$$

and

(4) $$log_e\left(\frac{H}{M}\right) = \frac{t(\alpha-1)}{\alpha\tau}$$

After conversion to log_2 and reintroducing $t_{½}$, equation 4 becomes

(5) $$log_2\left(\frac{H}{M}\right) = \frac{1}{log_e 2} \cdot \frac{t(\alpha-1)}{\alpha\tau} \approx 1.443 \cdot \frac{t(\alpha-1)}{\alpha \cdot 1.443 \cdot t_{½}}$$

And finally

(6) $$log_2\left(\frac{H}{M}\right) = \frac{t(\alpha-1)}{\alpha t_{½}}$$

Appendix Figure S1

H/L ratios are highly repeatable. Comparisons of H/L ratios (expressed as $\log_2$(H/L)) derived for 1409 proteins in two separate experiments for untreated (**A**) and lactacystin treated (**B**) cells. Note the excellent reproducibility for the majority of proteins.

Appendix Figure S2

Comparison of half-life estimates with previously published estimates. Rough half-life estimates based on a single H/L measurement at 24 hours compared to previously published half-life estimates based on four time points (0,1,3,7 days; Cohen *et al*, 2013). Analysis was limited to proteins with published half-lives that did not exceed 15 days, resulting in comparisons of 1,335 proteins. See Materials and Methods for an explanation on the manner by which half-lives were estimated from H/L ratios at t=24h. Full data set is provided in Table EV2.

Appendix Figure S3

Correlation between average Log_2(H/M) values measured in the 3 experiments in which lactacystin was used, and average Log_2(H/M) values measured in the 2 experiments in which epoxomicin was used. Only proteins for which statistically significant changes were observed at 24 hours are shown (158 proteins). The correlation, while positive (r = 0.501) and significant ($P<10^{-14}$), was not perfect. The residuals probably reflect the fact that H/M ratios were derived from only 3 and 2 experiments as well as the small dynamic range of $\log_2$H/M changes (-0.5 to 1.0). In addition, the specificities and efficacies of the two inhibitors are not identical; consequently we cannot rule out the possibility that the slower degradation rates observed for a number of proteins in the presence of lactacystin is due to inhibition of proteases other than the proteasome which are not inhibited by epoxomicin. Gray arrows point to (left to right) RGD1311575, Add3, Ppp1r9a/neurabin-1.

Appendix Figure S4

Effects of lactacystin on spontaneous network activity levels. Cortical neurons were grown on multielectrode (MEA) substrates for 17 days in an identical fashion to preparations used for SILAC experiments, and then mounted on an MEA amplifier. Spontaneous activity was measured for 5 hours after which lactacystin was added to the MEA dishes to a final concentration of 10µM. Recordings were then continued for another 20 hours. Action potentials (spikes) measured from all 60 electrodes were

accumulated at 1 min intervals (open circles), and the resulting spike rates were averaged over one hour time windows (closed circles). All spike rates are expressed as spikes/sec per electrode. Three separate experiments. Some variability was observed between networks, but lactacystin did not seem to exert any particular effects.

Appendix Figure S5

Verifying the activity of inhibitor stocks. Networks of cortical neurons in culture were exposed to 10µM lactacystin or 5µM epoxomicin at DIV 14. The cells were harvested after indicated periods, and examined by Western blots. Inhibitor activity was confirmed by verifying an accumulation of ubiquitin conjugates assayed by labeling with an anti-Ub-conjugate antibody. See Materials and Methods for further details.

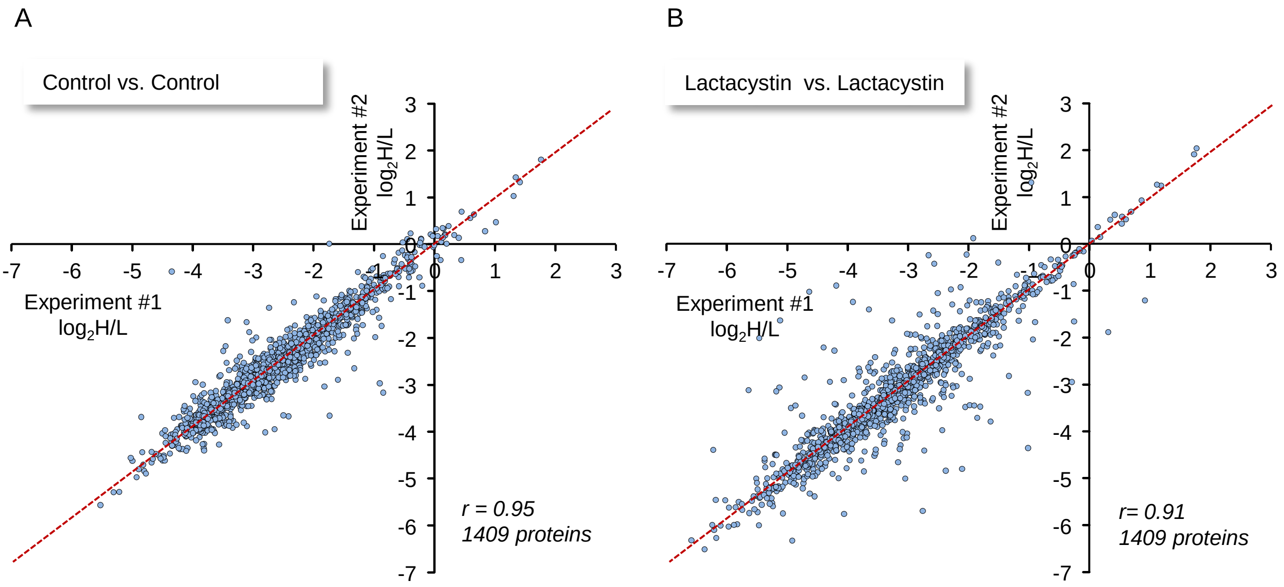

Appendix Figure S1

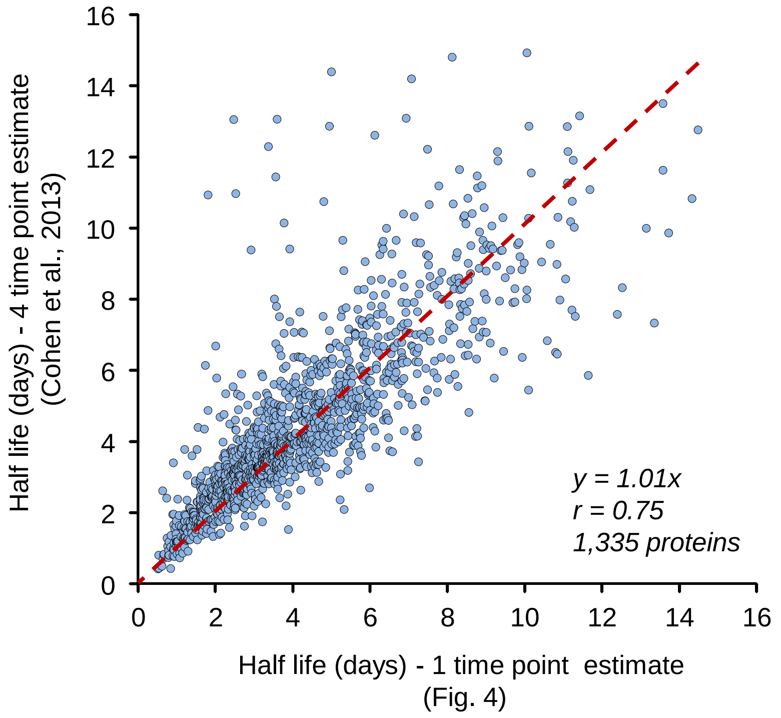

Appendix Figure S2

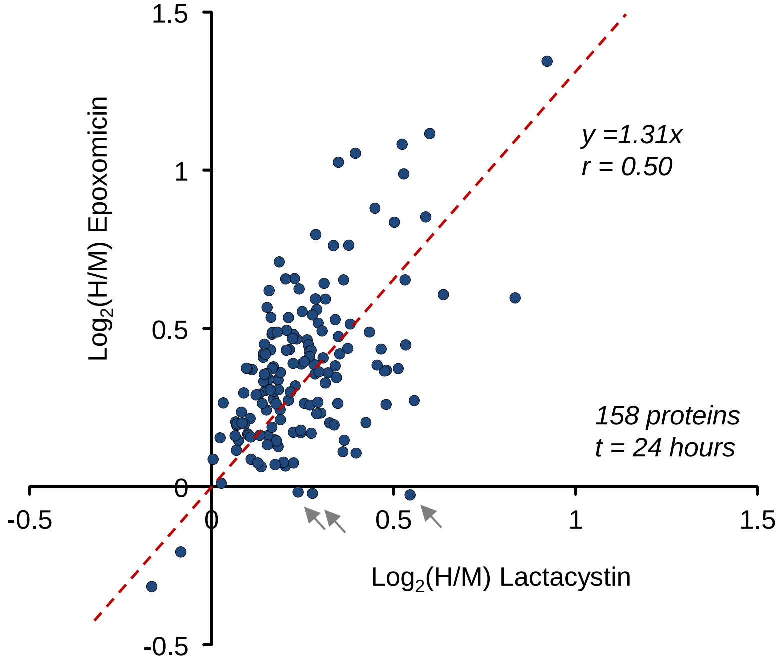

Appendix Figure S3

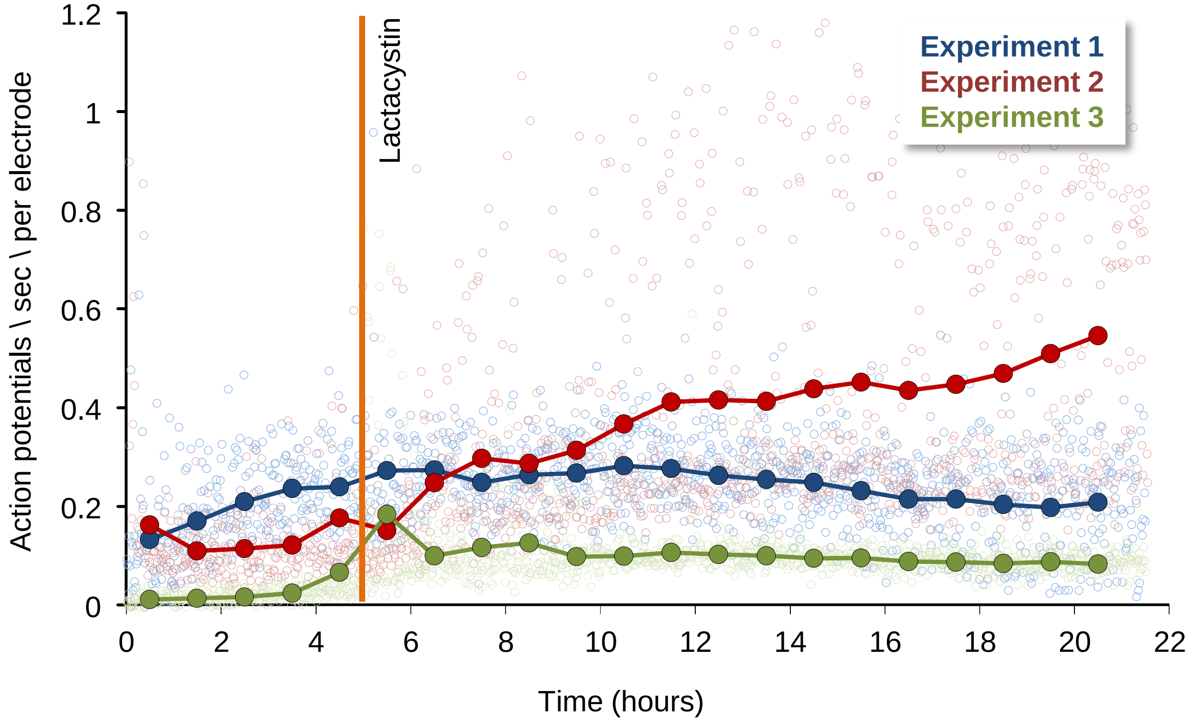

Appendix Figure S4

Time in Lactacystin (hours) 0 2 4 6 24

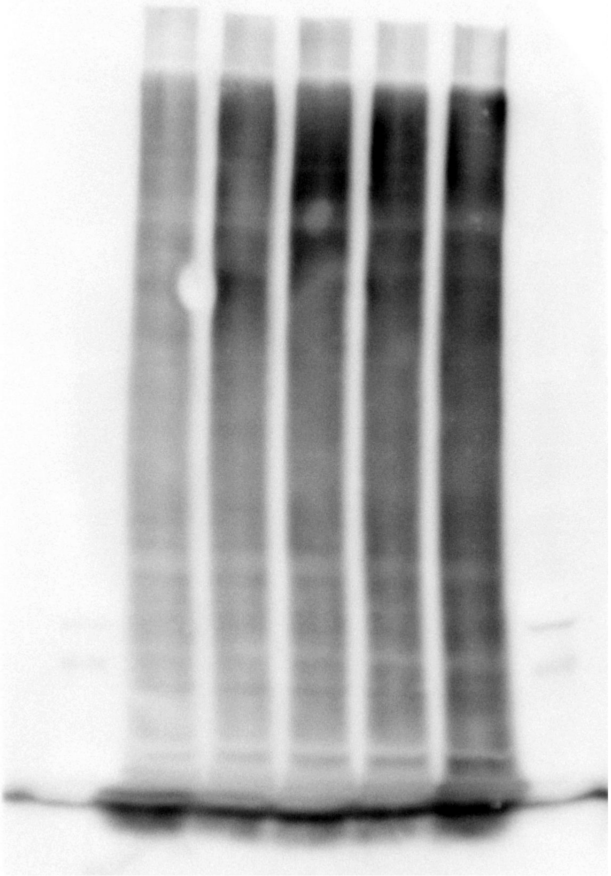

Appendix Figure S5
