# Supplementary material for: The effects of proteasomal inhibition on synaptic proteostasis
Source: EMBO J. 2016 Sep 9;35(20):2238–62. doi: 10.15252/embj.201593594 (PMC5069550; doi:10.15252/embj.201593594)
Supplement: Supplementary file 2 — Expanded View Figures PDF [file EMBJ-35-2238-s002.pdf]

Expanded View Figures

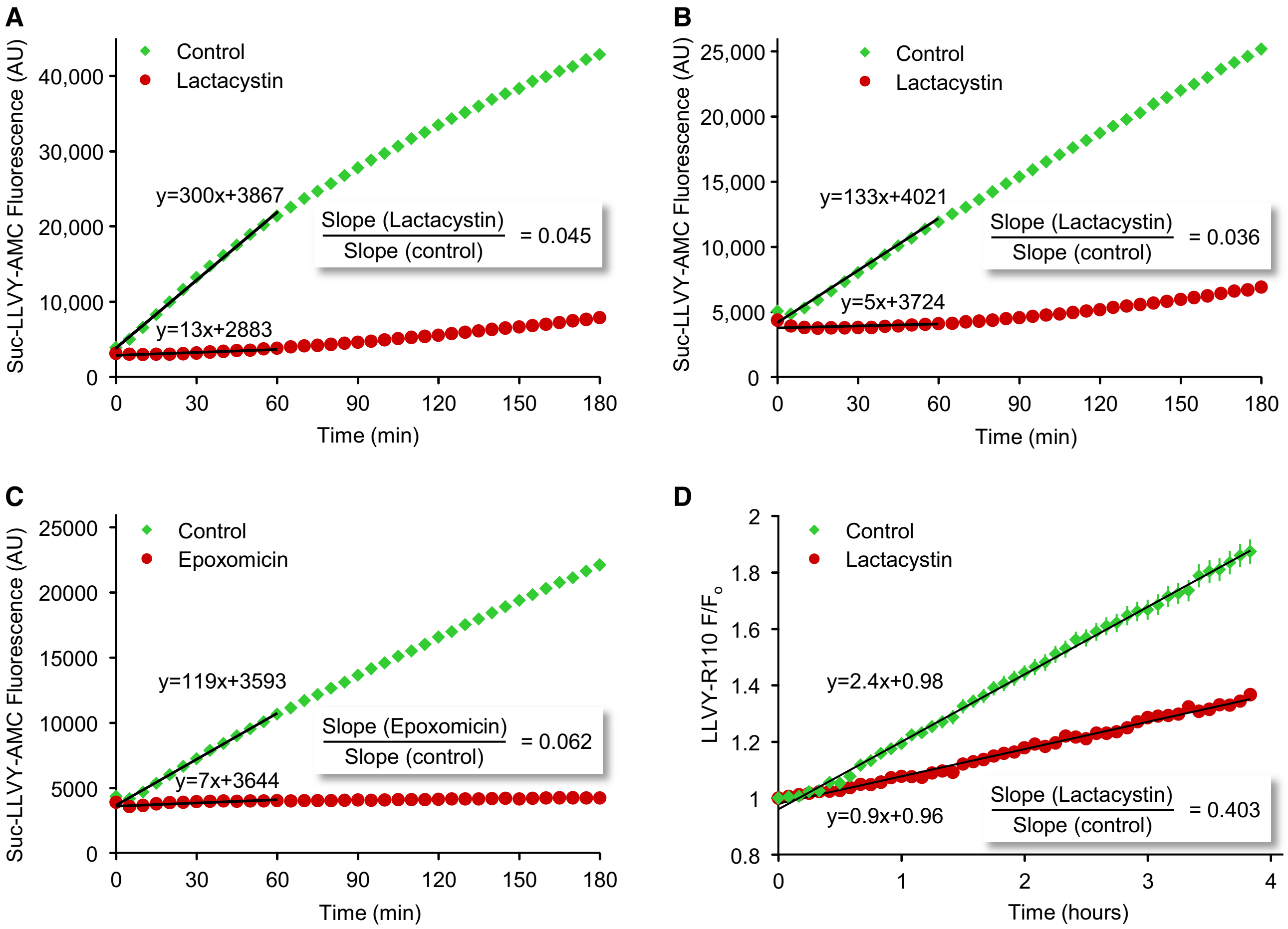

Figure EV1. Measuring proteasomal inhibition using fluorogenic substrates.

A, B Cortical neurons grown for 2 weeks in culture were treated with lactacystin (10 μM) or carrier solution for 4 h, washed vigorously with PBS after which they were extracted, and assayed for proteasomal (chymotrypsin-like) proteolytic activity using the fluorogenic substrate Suc-LLVY-AMC (see Materials and Methods for further details). Linear fits to the first hour are shown as black lines. Fit parameters are displayed next to lines. Two separate experiments. *Y*-axis, arbitrary fluorescence units.

C Same as in (A) and (B) except that cells were treated here with epoxomicin (5 μM).

D Proteasomal inhibition measured with fluorogenic substrates in living neurons. Cortical neurons were treated with lactacystin (10 μM) or carrier solution for 4 h, after which they were exposed to LLVY-Rhodamine-110 and followed by time-lapse microscopy. Fluorescence was thereafter measured in neuronal cell bodies of neurons. Note that the fluorescence began to increase only after ~30 min. Linear fits are shown as black lines. Fit parameters are displayed next to lines. Average and SEM for 121 cells from four experiments.

 © 2016 The Authors

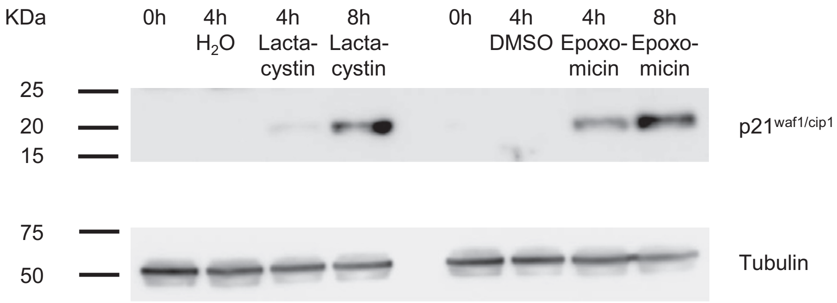

Figure EV2. Proteasome inhibitors drive rapid accumulation of $p21^{waf1/cip1}$.

Western blots prepared from extracts of cortical neurons in culture and probed with antibodies to $p21^{waf1/cip1}$ (top) or tubulin (bottom). Cells were untreated or treated with lactacystin (10 μM), epoxomicin (5 μM), H_2O or DMSO for 4 or 8 h as indicated.

Source data are available online for this figure.

© 2016 The Authors

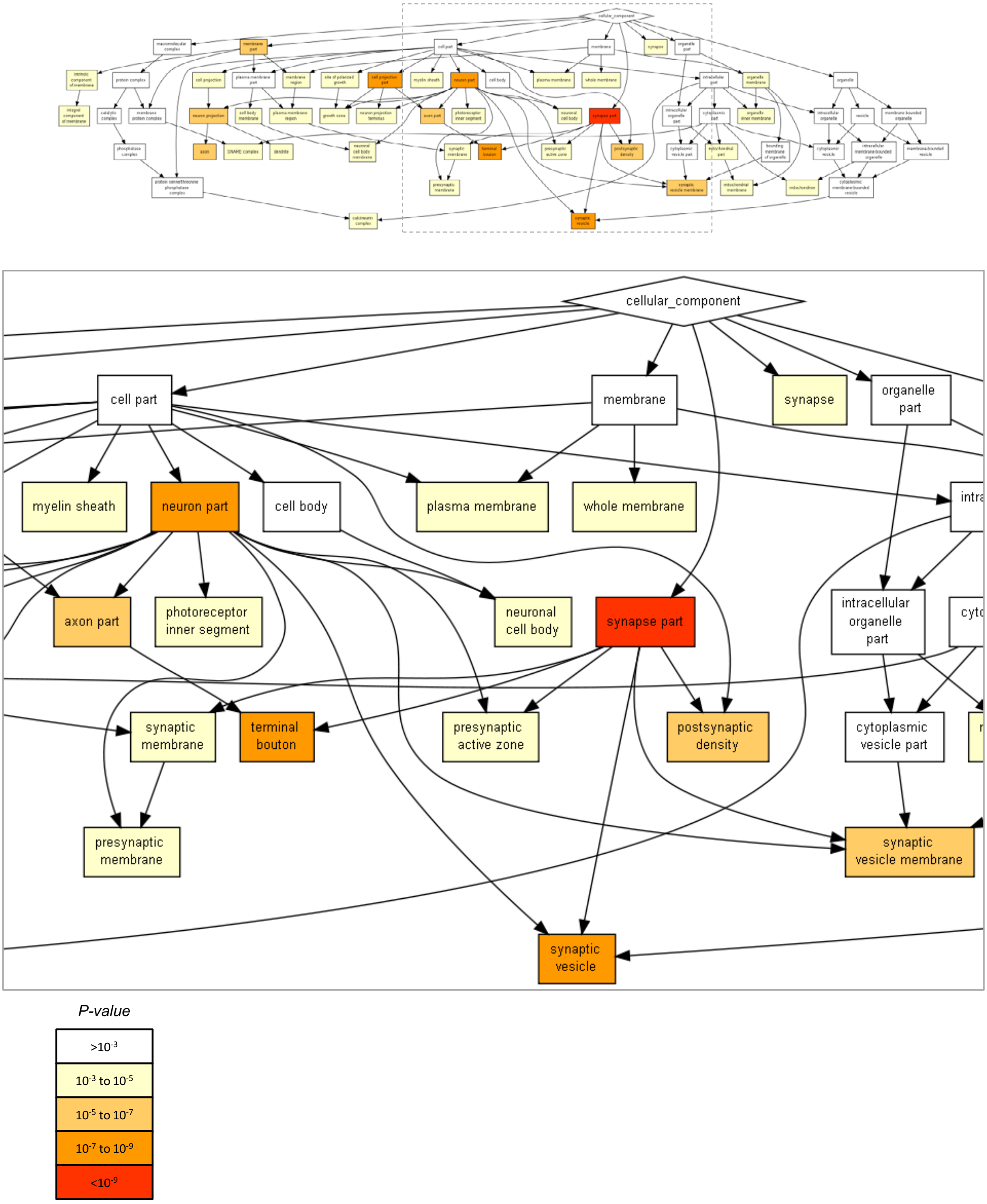

Figure EV3. Enrichment analysis of proteins whose H/L ratios were reduced following 24 h in the presence of lactacystin.
Proteins were sorted according to fold changes in the H/L ratios $\left(\frac{H/L_{lactacystin}}{H/L_{control}}\right)$ in the experiments described in Fig 3 (expressed as $D = \log_2(H/L)_{Lactacystin} - \log_2(H/L)_{control}$) and subjected to GO-based enrichment analysis using the public domain tool “GORILLA” (Gene Ontology enRIchment anaLysis and visuaLizAtion tool). The list included 1,362 proteins for which (i) D was negative, (ii) D was positive but within 3 standard deviations of the variability observed in the two control experiments data (i.e. $\log_2(H/L)_{control\_exp1} - \log_2(H/L)_{control\_exp2}$), that is, $D < 0.95$. The top panel shows the full diagram (category = “cellular component”), and the bottom panel shows an enlarged version of the diagram’s center. The statistical significance of enrichment scores (minimum hypergeometric score; calculated by GORILLA) is color-coded according to the index at the bottom.

 © 2016 The Authors

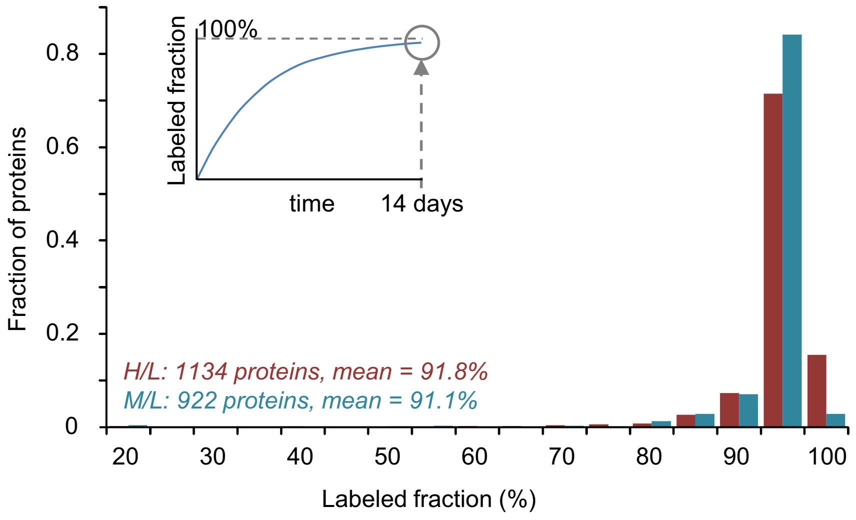

Figure EV4. Saturation of labeling with labeled amino acids.
Distribution of labeling saturation (illustrated in inset) following 2 weeks of growth in media containing H- or M-labeled lysine and arginine. Labeling saturation was measured by quantifying the H/L and M/L ratios and converting these into labeling saturation values as detailed in Materials and Methods. Data obtained from one SILAC experiment for each sample.

© 2016 The Authors

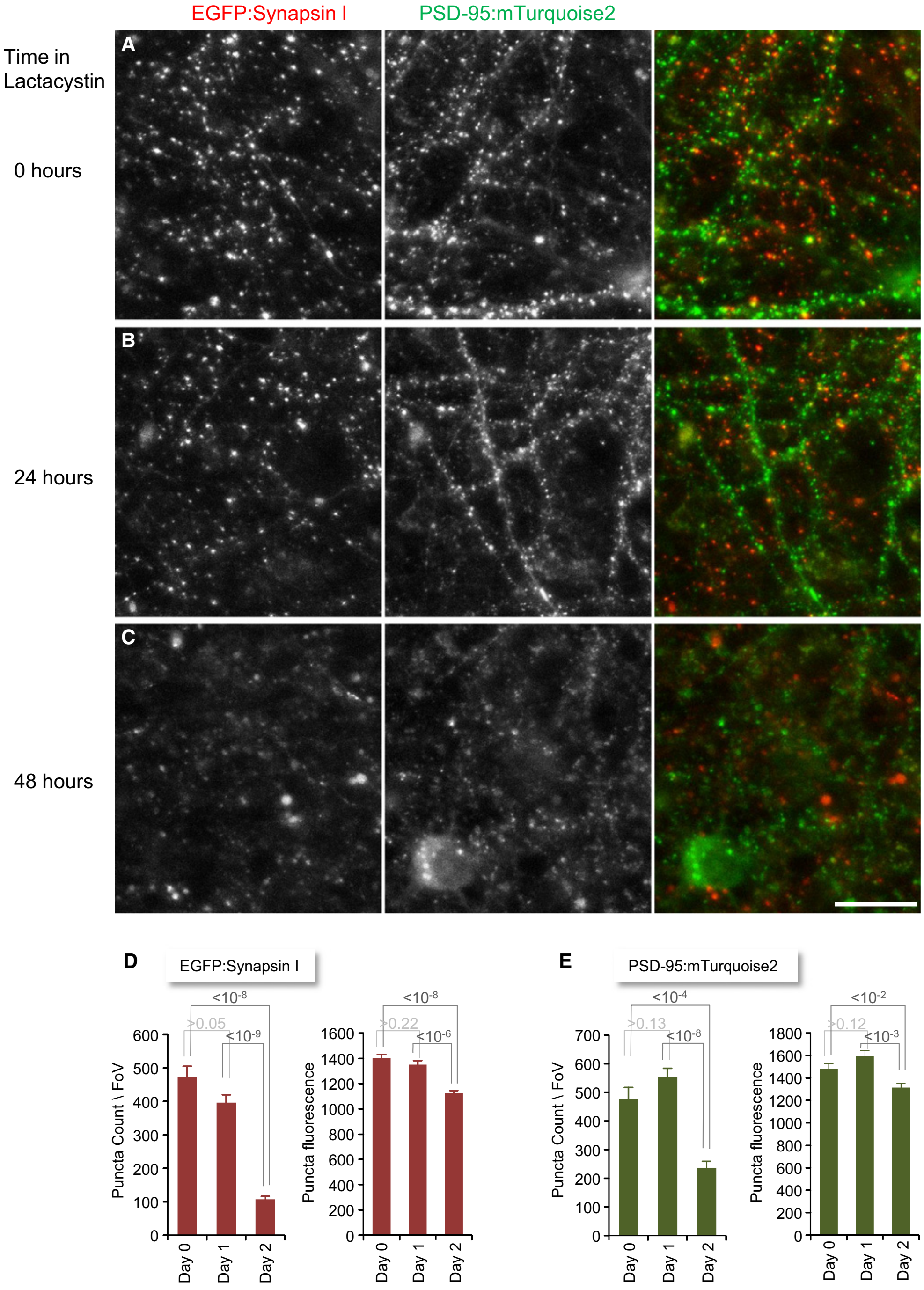

Figure EV5. Morphological features of synaptic connectivity following prolonged exposure to lactacystin.

A–C Cortical neurons, growing on glass-bottomed Petri dishes, were infected with lentiviral vectors driving the expression of the postsynaptic density protein PSD-95 fused to mTurquoise2 (PSD-95:mTurquoise2) and the presynaptic protein synapsin I fused to EGFP (EGFP:Synapsin I). At day 16 in culture, cells were exposed to lactacystin (10 μM) and imaged: (A) immediately, (B) after 24 h, and (C) after 48 h. No obvious changes in synapse density or other morphological features were observed after 24 h, but signs of change were very evident after 48 h. Scale bar, 20 μm.

D Quantification of EGFP:synapsin I puncta counts per field of view (FoV) and average puncta fluorescence in each FoV. Averages and SEM for 16–17 FoVs per time point. *P*-values of comparisons (two-tailed Welch tests) are indicated above columns.

E Quantification of PSD-95:mTurquoise2 puncta counts per FoV and average puncta fluorescence in each FoV. Averages and SEM for 16–17 FoVs per time point. *P*-values of comparisons (two-tailed Welch tests) are indicated above columns.

Data information: All data in (D,E) collected in one experiment.

 © 2016 The Authors
