# Supplementary figures and images for: The effects of proteasomal inhibition on synaptic proteostasis
Source: EMBO J. 2016 Sep 9;35(20):2238–62. doi: 10.15252/embj.201593594 (PMC5069550; doi:10.15252/embj.201593594)

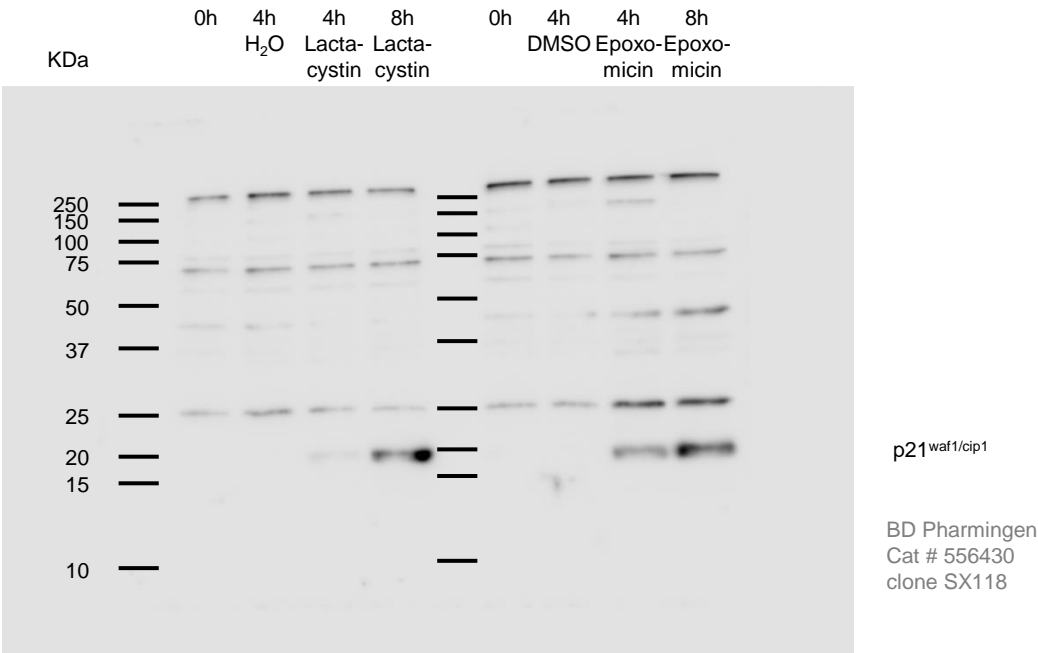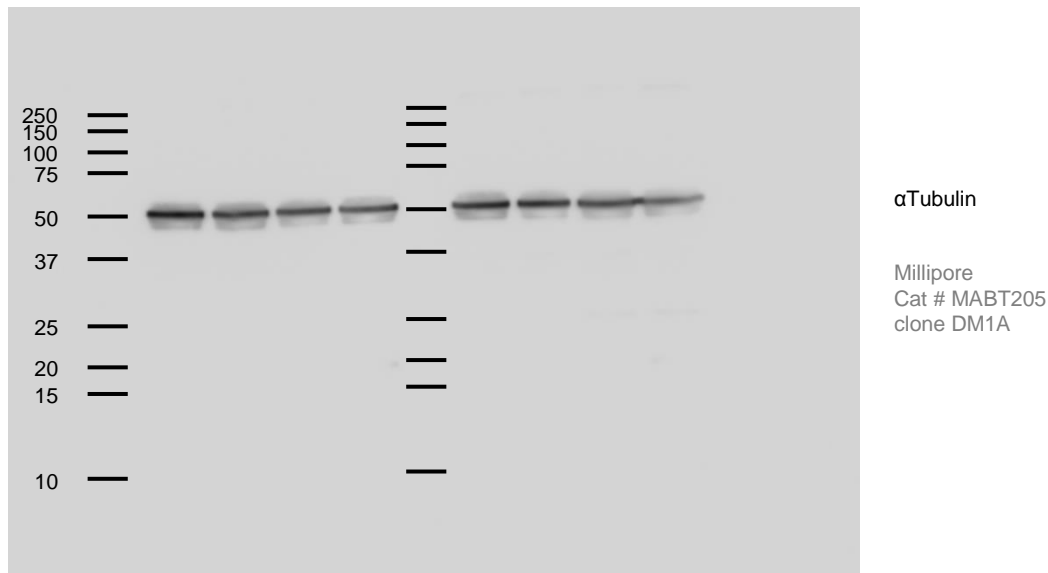

Supplement: Supplementary file 7 — Source Data for Expanded View [file EMBJ-35-2238-s007.zip › EMBOJ201593594R_SourceDataForFigureEV2.pdf]
